# Distance-based delivery of exercise for people treated for breast, prostate or colorectal cancer: a study protocol for a randomised controlled trial of EX-MED Cancer Sweden

**DOI:** 10.1186/s13063-023-07152-z

**Published:** 2023-02-17

**Authors:** Melissa Kotte, Kate A. Bolam, Sara Mijwel, Renske Altena, Prue Cormie, Yvonne Wengström

**Affiliations:** 1 Department of Neurobiology, Care Sciences and Society, Karolinska Institutet, Stockholm, Sweden; 2 Department of Physical Activity and Health, The Swedish School of Sport and Health Sciences, Stockholm, Sweden; 3 Department of Physical Performance, Norwegian School of Sport Sciences, Oslo, Norway; 4 Medical Unit Breast, Endocrine Tumors and Sarcoma, Theme Cancer, Karolinska University Hospital, Stockholm, Sweden; 5 Department of Oncology-Pathology, Karolinska Institutet, Stockholm, Sweden; 6 Peter MacCallum Cancer Centre, Melbourne, VIC Australia; 7 Sir Peter MacCallum Department of Oncology, The University of Melbourne, Melbourne, VIC Australia; 8 EX-MED Cancer, Melbourne, VIC Australia

**Keywords:** Exercise, Cancer, Fitness, Distance-based, Home-based

## Abstract

**Background:**

Regular exercise has been shown to have beneficial health effects in cancer survivors, including improving quality of life and other important health outcomes. However, providing people with cancer with easily accessible, high-quality exercise support and programs is a challenge. Therefore, there is a need to develop easily accessible exercise programs that draw upon the current evidence. Supervised, distance-based exercise programs have the benefit of reaching out to many people whilst providing the support of an exercise professional. The aim of the EX-MED Cancer Sweden trial is to examine the effectiveness of a supervised, distance-based exercise program, in people previously treated for breast, prostate, or colorectal cancer, on health-related quality of life (HRQoL), as well as other physiological and patient-reported health outcomes.

**Methods:**

The EX-MED Cancer Sweden trial is a prospective randomised controlled trial including 200 people that have completed curative treatment for breast, prostate, or colorectal cancer. Participants are randomly allocated to an exercise group or a routine care control group. The exercise group will participate in a supervised, distanced-based exercise program delivered by a personal trainer who has undertaken specialised exercise oncology education modules. The intervention consists of a combination of resistance and aerobic exercises with participants completing two 60-min sessions per week for 12 weeks. The primary outcome is HRQoL (EORTC QLQ-C30) assessed at baseline, 3- (end of intervention and primary endpoint) and 6-months post-baseline. Secondary outcomes are physiological (cardiorespiratory fitness, muscle strength, physical function, body composition) and patient-reported outcomes (cancer-related symptoms, fatigue, self-reported physical activity), and self-efficacy of exercise. Furthermore, the trial will explore and describe the experiences of participation in the exercise intervention.

**Discussion:**

The EX-MED Cancer Sweden trial will provide evidence regarding the effectiveness of a supervised, distance-based exercise program for survivors of breast, prostate, and colorectal cancer. If successful, it will contribute to the implementation of flexible and effective exercise programs as part of the standard of care for people following cancer treatment, which is likely to contribute to a reduction in the burden of cancer on the individual, health care system and society.

**Trial registration:**

www.ClinicalTrials.gov NCT05064670. Registered on October 1, 2021.

## Background

There is now robust evidence showing that both aerobic and resistance exercise plays an important role in improving the quality of life, psychological health, and physical function, as well as helping to manage common cancer-related side effects, among people who have completed cancer treatment [1–5]. International health organisations are therefore calling for exercise to be incorporated into routine cancer care [6–8]; however, the great challenge in the field of exercise oncology is being able to provide people with cancer easily accessible, high-quality exercise support and programs. Consequently, there is an urgent need to develop exercise programs that draw upon the current evidence base that can be delivered to as many people as possible.

In recent years, particularly in light of a major pandemic, there has been an increase in distance-based delivery of health care in an attempt to improve supportive care services for people with cancer [9, 10]. Interventions such as symptom monitoring and management, remote rehabilitation program delivery, nursing support, health education, and psychotherapy are provided using digital technologies including telephones or smartphones, video, and web-based portals [9, 11]. Although much of health care delivery still occurs face-to-face, distance-based interventions have been shown to improve health-related outcomes, such as health-related quality of life (HRQoL), functional capacity, and cancer-related symptoms [9, 11]. Additionally, distance-based interventions are now much more accepted, are clinically safe, and have the major advantage of reducing the impact on a person’s daily activities, for example by minimising the burden of travel [12–14].

The original EX-MED Cancer program (exercise medicine program for people with cancer) [15], a supervised, facility-based exercise program, was developed, trialled, and implemented in Australia to incorporate best practice evidence-based exercise prescription whilst minimising burden to patients and health care professionals [16]. It capitalises on existing community-based workforce and resources, to facilitate widespread participation by cancer survivors, scalability, and sustainability of the program with the goal of embedding exercise medicine into routine cancer care. EX-MED Cancer Sweden has been adapted from the Australian model to a distance-based exercise program. Supervised, distance-based exercise programs have the benefit of reaching out to as many people as possible, allowing participants to exercise at home, avoiding public places and travel, whilst still gleaning the benefits of supervision by a trained fitness professional [17, 18]. Additional commonly identified facilitators of exercise among cancer survivors include group-based exercise with other cancer survivors, and exercise that is individually tailored, varied, and gradually progressive with individual feedback [17, 18].

Whilst the EX-MED Cancer program has been successfully implemented in Australia, no similar program exists or has been trialled in Sweden. EX-MED Cancer Sweden is a single-centre phase 3 randomised controlled trial. It aims to examine the effectiveness of a supervised, distance-based exercise program, in people previously treated for breast, prostate, or colorectal cancer, on HRQoL, as well as other physiological and patient-reported health outcomes. Additionally, we aim to explore and describe the experiences of participation in the exercise program, to inform further design and implementation of exercise programs for cancer survivors.

## Methods and design

**Fig. 1:**
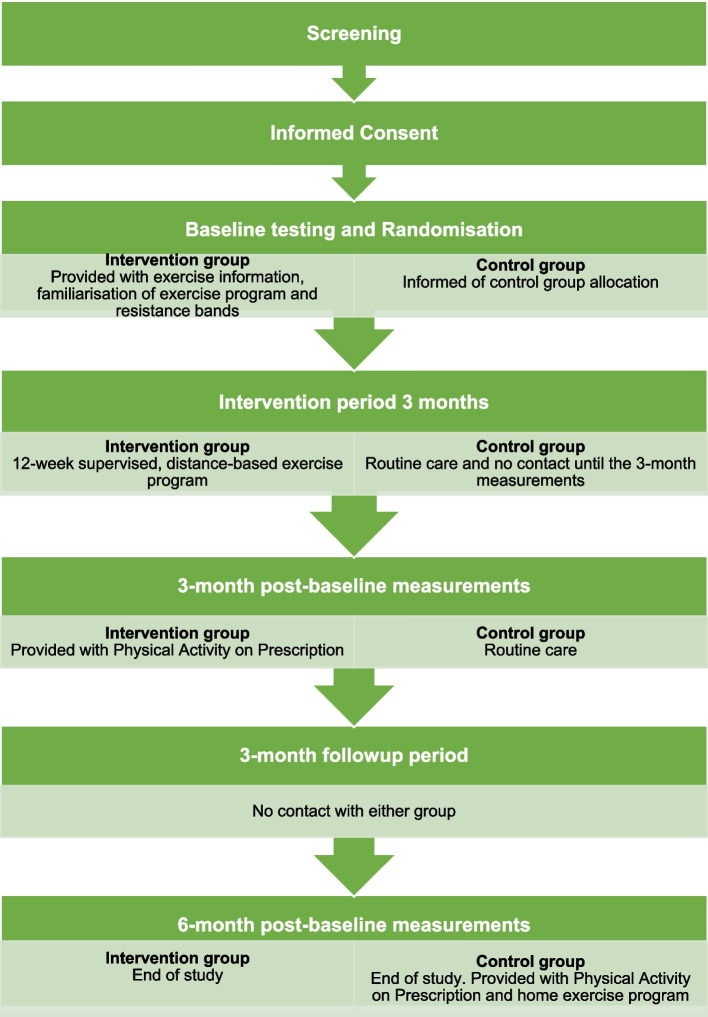
Schematic flow diagram of the EX-MED Cancer Sweden trial

EX-MED Cancer Sweden is a prospective superiority two-armed single-centre randomised controlled trial, testing a supervised, distance-based physical exercise intervention for survivors of breast, prostate, or colorectal cancer (Table 1 and Fig. 1). The trial is based on the EX-MED Cancer trial, ongoing program and professional development course originally developed and implemented in Australia [15, 16, 19]. The primary outcome is HRQoL. Secondary outcomes are physiological (cardiorespiratory fitness (CRF), muscle strength, physical function, body composition) and patient-reported outcomes (cancer-related symptoms, fatigue, self-reported physical activity), and self-efficacy of exercise. Furthermore, the trial will explore and describe the experiences of participation in the exercise intervention.

**Table 1:**
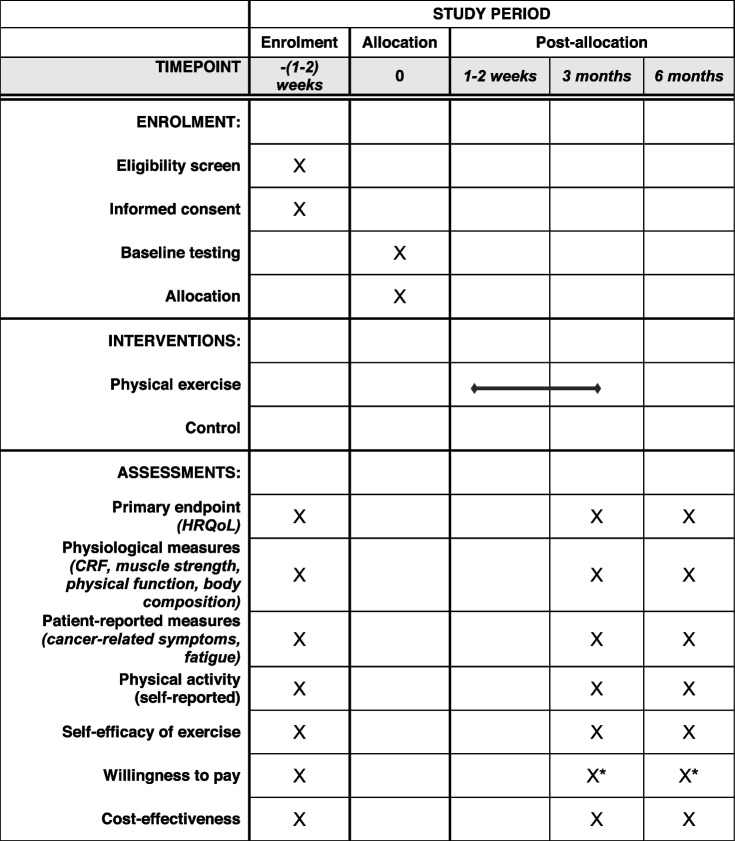
Schedule of enrolment, interventions, and assessments for the EX-MED Cancer Sweden trial

*HRQoL* Health-related quality of life, *CRF* Cardiorespiratory fitness

*Only for the exercise group

### Study setting

The exercise lab at The Karolinska Comprehensive Cancer Centre, Stockholm, Sweden, is responsible for the coordination of EX-MED Cancer Sweden. It is the central point of contact for study participants and health care professionals and where testing will be conducted. Study participants will exercise in their own homes, or other suitable locations, using the online platform Microsoft® Teams, where a personal trainer will be the host and supervisor for the session.

### Population

People diagnosed with any type of stage I–IIIa breast, prostate, or colorectal cancer who have undergone and completed curative treatment in the last five years whilst fulfilling all inclusion criteria are eligible to participate in the trial (Table 2). People are eligible if they are currently receiving or are scheduled to receive anti-hormonal therapy. Informed consent is required before baseline testing and will be obtained by a nursing member of the research team.

**Table 2:** Inclusion and exclusion criteria in the EX-MED Cancer Sweden trial

Inclusion criteria	Exclusion criteria
• Age ≥ 18 years • Diagnosed with any type of stage I–IIIa breast, prostate or colorectal cancer • Undergone and completed curative treatment (eligible if currently receiving/scheduled to receive anti-hormonal therapy) within the last 5 years at the Karolinska Comprehensive Cancer Centre, Sweden	• Currently receiving or scheduled to receive cancer treatment (except anti-hormonal therapy) • Any medical conditions that may prevent safe participation in the testing or exercise demands of the study [20] • Performing regular exercise throughout the last month defined as undertaking at least 150 min moderate-intensity aerobic exercise and two or more structured resistance exercise sessions per week • Unable to read and speak Swedish

A selection of people participating in the exercise intervention and the personal trainers, employed at the gym franchise SATS, will be recruited to a qualitative study exploring and describing experiences of participation in the intervention.

### Intervention

All participants will undergo baseline testing of physical fitness and strength prior to randomisation. Participants randomised to the intervention will be registered for the next exercise group to commence within 2 weeks of baseline testing. Participants will complete two 60-min distance-based exercise sessions per week for 12 weeks (24 sessions in total). The program consists of a combination of resistance and aerobic exercises. The exercise sessions are delivered by upskilled personal trainers in groups of up to eight people. Educational modules have been developed for the personal trainers and are delivered by the research group prior to delivering the intervention. These modules are the EX-MED Cancer PD course [19], developed in Australia by author PC, with modifications made to contextualise the content to the Swedish healthcare setting. Modules consist of an introduction to cancer, side effects of cancer treatments, screening and monitoring of cancer, exercise prescription for cancer and working with people with cancer. Prior to commencing the exercise intervention, participants will receive an in-person familiarisation of the exercise program from a member of the research team. They will be provided with a hard copy of the exercise program, resistance bands for training, a detailed information booklet about exercise for people with cancer, and a logbook to record attendance, exercise adherence and level of exertion. Any barriers to exercise identified by the participant or the research team will also be discussed, and solutions developed together.

The exercise intervention is a progressive circuit training program consisting of a consecutive series of timed exercises performed one after the other. Incorporating whole-body functional exercises, sessions include four blocks with three exercises per block, including two whole-body resistance-focused and one aerobic-focused. Each set of three exercises will be performed for a prescribed amount of time with the participant completing as many repetitions as possible in this time. Each exercise is followed by a short rest interval with a longer rest interval after each set. Each set will be repeated three times before moving on to the next block containing three new exercises. Intensity will be manipulated by using body weight and two different resistance bands, light and medium, and by the participant increasing or decreasing the number of repetitions performed during the prescribed time. Each session will include a warmup and cooldown. The personal trainer will monitor each participant during the sessions to ensure exercises are performed correctly and safely. Exercise prescription is progressive and will be modified by the personal trainer according to individual response. The personal trainer will support the participants to achieve an overall exercise intensity of 14–18 on Borg’s Rating of Perceived Exertion (RPE) scale. Participants will be encouraged to perform at least 150 min of moderate physical activity per week, inclusive of the exercise intervention sessions.

Following the completion of the exercise intervention, immediately after the completion of the post-intervention testing session, participants will receive a written Physical Activity on Prescription (PAP) and be encouraged to continue to exercise. The individualised PAP is a written prescription provided by a registered healthcare professional that prescribes physical activity outside of healthcare services, with the aim of increasing levels of physical activity and integrating it into everyday life [21]. They will be given the opportunity to discuss any goals, preferences, or barriers to future exercise. Participants will not receive any contact or exercise support in the 3-month follow-up period following the initial 3-month intervention.

### Control

As the aim of the trial is to compare the effectiveness of the exercise program with routine care, the control group is a routine care control group. This means that study participants will receive routine care according to national guidelines [22], which does not involve a standardised, structured exercise program, but may include information and advice about physical activity from the treating nurse, physician or physiotherapist.

At the completion of the trial, following the 6-month post-baseline (follow-up) testing session, the study participants in the control group will be offered a home exercise program, a written PAP, and the same exercise information booklet the exercise intervention group was given. In addition, they will also be offered a hard copy of the same exercise program used in the intervention, including pictures and instructions for the exercises.

### Outcomes

Participants will complete measurements for primary and secondary endpoints at baseline, 3 months post-baseline (post-intervention), and 6 months post-baseline (follow-up) (Table 1). Participants will attend the exercise lab at the Karolinska Comprehensive Cancer Centre for the testing sessions and questionnaires will be completed online.

#### Primary outcome

The primary endpoint, patient-reported HRQoL at 3 months (post-intervention), will be assessed using the European Organization for Research and Treatment of Cancer quality of life questionnaire (EORTC QLQ-C30), a cancer-specific instrument that measures HRQoL in relation to physical, emotional, social, cognitive, and everyday function [23, 24].

#### Secondary outcomes

1. Estimated CRF (estimated/predicted maximal oxygen uptake, VO_2_max) assessed by the Ekblom-Bak submaximal cycle test [25, 26] on a cycle ergometer (Monark 928E, Monark Exercise AB, Sweden).
2. Muscle strength assessed by the hypothetical 1 repetition maximum (1-RM) test for both the upper body (chest press) and lower body (leg press) [27]. Additionally, handgrip strength [28] assessed using a hydraulic hand dynamometer (SAEHAN Corporation, South Korea).
3. Physical function assessed by the 5 sit-to-stand test, where participants will be asked to stand up and sit down in a chair as fast as they safely can five times [29].
4. Body composition measured using bio-impedance analysis (BIA) to assess body fat (BF%), fat mass (kg) and lean mass (kg), using the (InBody770, InBody Corporation, South Korea).
5. Common cancer-related symptoms assessed by the 32-item Memorial Symptom Assessment Scale (MSAS) including occurrence, frequency, severity, and distress associated with each symptom using four- and five-point rating scales [30]. The instrument has been validated for Swedish patients with breast cancer [31].
6. Fatigue assessed by the Piper Fatigue Scale including the four dimensions of behavioural/severity, affective meaning, sensory, and cognitive/mood [32]. The instrument has been shown to be reliable among Swedish populations of patients with cancer [33].
7. Self-reported physical activity assessed by the Modified Godin Leisure Time Physical activity questionnaire [34].
8. Patient-reported HRQoL will be also measured at the follow-up timepoint as a secondary endpoint.
9. Self-efficacy for exercise (SEE) assessed by the Self-Efficacy of Exercise questionnaire [35] – Swedish version (SEE-SV) [36, 37] to examine whether SEE is a predictor of short and long-term changes in exercise behaviour in this trial.

Attendance and adherence to the exercise sessions will be continuously reported by the personal trainer to the research team. Additionally, cost-effectiveness will be measured using the EuroQol 5 Dimension 5 Level (EQ-5L-5D) questionnaire [38] and purpose-designed cost-effectiveness and willingness to pay questionnaires.

To explore and describe the experiences of participation in the exercise program, the participants in the intervention group will complete a purpose-designed questionnaire. Furthermore, a selection of the participants from the intervention group and the personal trainers involved in delivering the exercise program will be invited to participate in separate focus groups or individual interviews.

Participant demographics including general medical and cancer history, resting heart rate, resting blood pressure, body mass, height, body mass index, age, sex, smoking status, education level, relationship status, and percentage of sick leave will be recorded to characterise the participant groups.

### Participant timeline

Following screening and providing informed consent, participants will answer questionnaires online and complete baseline testing at the exercise lab (Table 1 and Fig. 1). Following the baseline testing session participants will be randomised to either the intervention group or routine care control group. The intervention group will commence the exercise program within 2 weeks of baseline testing. The control group will continue to receive usual care and will have no contact with the research team until the 3-month testing session, unless initiated by the participant. Participants in both groups will answer questionnaires and complete testing sessions again at 3 months post-baseline (post-intervention) and 6 months post-baseline (follow-up). There will be no contact with the research team for either group between the 3- and 6-month post-baseline unless initiated by the participant.

### Sample size

We aim to include 200 participants in this trial. Sample size calculations have been based on having sufficient power to detect a medium effect size (Cohen’s *d* = 0.5) for the primary endpoint, overall patient-reported HRQoL post-intervention. We regard an improvement in HRQoL in the intervention arm from baseline to three months post-baseline relative to the control arm to be of relevance. With a power of 80% and an alpha of 5% (two tailed) from pre- to post-intervention, a total of 160 participants needs to be included; 80 in each arm.

To ensure sufficient participant numbers at the completion of the trial, sample size calculations account for a 20% attrition rate, leading to a total sample size of 200 participants.

### Recruitment

Recruitment commenced in January 2022 and is ongoing. Participants are recruited from Karolinska Comprehensive Cancer Centre, Sweden using the following three methods:

1. Nurses and physicians involved in the care of the respective diagnosis groups will inform potential participants about the trial during a regular visit or by mail/letter or telephone. Details of interested individuals will be forwarded to the research team.
2. A nurse in the research team will review electronic patient records to identify potential participants and contact the treating nurse or physician to obtain permission to contact the individual.
3. Information posters and brochures will be placed in the waiting rooms of the respective outpatient clinics where patients are followed up so that interested individuals can make direct contact with the research team.

Interested individuals will be provided with verbal and written information about the trial by a nursing member of the research team who will also screen for eligibility. Screening will be conducted in consultation with the trial physician in the case of any individual having a medical condition that may prevent safe participation in the study. Eligible individuals will then be provided with the full participant information form explaining the trial aims and procedures. After reading through the full information form and having the opportunity to ask questions, participants will be invited to sign a written informed consent form by a nursing member of the research team.

The personal trainers invited to participate in the focus groups or individual interviews will be provided with the full participant information form, given the opportunity to ask questions, and invited to sign a written informed consent form.

### Assignment of interventions

After the completion of baseline testing, study participants will be randomised by a member of the research team on a 1:1 basis in block samples, using the computer-generated randomisation program ALEA. Randomisation will be stratified by disease type (breast, prostate or colorectal) and treatment with chemotherapy (yes or no). Study investigators and participants will be blinded to group allocation at baseline, but not at the subsequent testing timepoints. The design is open label so unblinding will not occur.

### Data collection and management

Data are collected and entered exclusively by members of the research team. In the interest of standardising the testing procedures, a standardised operating procedures manual has been developed and all testers will undergo standardised training to conduct the tests. The same members of the research team monitor the participants to ensure follow-up completion, including email and SMS reminders. All hard copies of forms are kept in a locked filing cabinet at Karolinska Institutet. All recorded data are pseudonymised. Participants will not be identified in the resulting manuscripts and reports. Only the study investigators have access to trial information and the dataset.

The EX-MED Cancer Sweden trial is registered at www.ClinicalTrials.gov (NCT05064670). Ethical permissions have been obtained by the Swedish Ethical Review Authority (2019–04,151, 2021–01,715). All participants must provide written informed consent to participate in this trial.

### Statistical methods

Analyses will be performed using an intention-to-treat approach. For the quantitative analysis, descriptive statistics will be used to describe baseline characteristics. Analysis of covariance (ANCOVA) and linear mixed modelling will be used to detect any significant differences between the two groups at three and six months, respectively. Corresponding effect sizes will also be calculated. Explorative sub-analysis by cancer type will be conducted to investigate if any effects of the exercise intervention are cancer type specific.

For the qualitative analysis, focus group discussions/individual interviews will be transcribed verbatim and analysed using qualitative content analysis.

### Serious adverse events

All (serious) adverse events ((S)AE) related to the study measurements or exercise, reported by the participant, observed by the personal trainer or study investigators will be recorded. Participants in both groups will be asked by a study investigator about any study measurement- or exercise-related (S) AEs systematically and in a standardised manner at the 3- and 6-month testing timepoints. Additionally, participants in the intervention group will be asked by the personal trainer, before and after each exercise session, about any potentially exercise-related (S) AEs that have occurred during or between exercise sessions and will report back to the study investigators. In the event of a SAE event during the exercise sessions, the personal trainer will immediately call for medical assistance and contact the study investigators. (S) AEs are reported to the participant’s physician or nurse in a standardised manner. Participants involved in the trial are covered by the national patient insurance system.

## Discussion

Exercise is a proven strategy to alleviate treatment-related side-effects and to maintain or improve HRQoL in cancer survivors [1–5]. Exercise rehabilitation strategies are therefore recommended within cancer care [6–8]. By providing a supervised, distanced-based exercise program we can reach out to more cancer survivors who will receive support and guidance from an exercise professional [17, 18]. To date, there are no standardised exercise programs for cancer survivors in Sweden, which is the underlying rationale for the randomised EX-MED Cancer Sweden trial.

EX-MED Cancer Sweden is an innovative, supervised distance-based approach to providing exercise support to cancer survivors in their own homes. Several aspects of the trial have contributed to its innovative nature. The exercise program is initiated through the hospital system but delivered by an upskilled community-based workforce. Exercise programs that include supervision are most effective [39], yet the health care system does not have the resources to provide every cancer survivor with supervised exercise programs. By upskilling exercise professionals in the community, we are creating an important bridge between the healthcare system and the community by providing more sustainable, yet high-quality, exercise support. Using a distance-based approach ensures the program is flexible, agile, and accessible. By reducing the travel burden for participants, we can reach out to more people, which increases the feasibility of the program. We need to capitalise on the increased use of distance-based technology to deliver healthcare interventions in this post-pandemic era [9, 10].

The design of the exercise program increases its’ feasibility and accessibility as participants do not require expensive training equipment that may not be available in the home environment. Additionally, distance-based training in a safe home environment [14] may lower the threshold for cancer survivors to start exercising. Personal trainers are also able to adapt and individualise exercises and exercise intensity can easily be increased or decreased. Such adaptions to the program ensure a personalised exercise prescription for each participant.

Contamination, where control participants adopt some sort of exercise, and drop-out among the control group are important considerations in exercise oncology trials as they can affect trial results [40, 41]. Meaningful improvements in control groups’ physical activity have been reported in 30% of physical activity intervention trials [40], and as such we will take measures to decrease the risk of contamination and drop-out [41]. Participants in the control group will not be permitted to see the exercise information booklet or the exercise program. Additionally, the control group will be promised a home exercise program at the completion of the trial.

In conclusion, in the EX-MED Cancer Sweden trial, we are examining the effectiveness of a supervised, distance-based exercise program, in people previously treated for breast, prostate, or colorectal cancer, on HRQoL, as well as other physiological and patient-reported health outcomes. Furthermore, we will explore and describe the experiences of participation in the exercise program. Knowledge gained from this trial will provide meaningful information needed to strengthen rehabilitation strategies within cancer care. If successful, EX-MED Cancer Sweden will contribute significantly to the national and international movement to implement flexible and effective exercise programs as part of standard of care for people following cancer treatment, which is likely to contribute to a reduction in the burden of cancer on the individual, health care system and society.

## Dissemination of results

The study investigators will inform all participants about the results of the study. The results of the study will be reported in peer-reviewed international journals, popular science articles, e.g., from patient organisations and professional associations, and presented at local and international conferences. Policymakers, healthcare providers, funding bodies, and other important stakeholders, e.g., patient organisations, will be informed on the outcomes of the EX-MED Cancer Sweden trial.

## Trial status

Protocol version number and date: version 2.0 date 11/12/2020.

Recruitment for the trial began in January 2022 and is expected to be completed by September 2023.

## Data Availability

The datasets used during the current study are available from the corresponding author on reasonable request.

## Abbreviations

HRQoL: Health-related quality of life
EX-MED Cancer: Exercise medicine program for people with cancer
EORTC QLQ-C30: The European Organization for Research and Treatment of Cancer quality of life questionnaire
VO_2_max: Estimated/predicted maximal oxygen uptake
1-RM: 1 Repetition maximum
CRF: Cardiorespiratory fitness
RPE: Rating of perceived exertion
PAP: Physical activity on prescription
BIA: Bio-impedance analysis
MSAS: Memorial Symptom Assessment Scale
SEE: Self-Efficacy of Exercise
SEE-SV: Self-Efficacy of Exercise – Swedish version
EQ-5L-5D: EuroQol 5 Dimension 5 Level
ANCOVA: Analysis of covariance
(S) AE: (Serious) adverse event
